# Neural network-based method for measuring the impacts of epileptic brain activities on cardiac cycles

**DOI:** 10.3389/fneur.2025.1555162

**Published:** 2025-07-30

**Authors:** Cândida H. L. Alves, Gilberto S. Alves, Rômulo Kunrath, Mariélia Barbosa L. de Freitas, João Pedro G. Castor, Allan Kardec Barros, Diego Dutra Sampaio, Jonathan Araújo Queiroz

**Affiliations:** ^1^Department of Psychology, Edufor Faculty, Idomed Faculty, São Luís, Maranhão, Brazil; ^2^Neuropsychiatry Unit, Nina Rodrigues Hospital, São Luís, Maranhão, Brazil; ^3^Department of Medicine I, Federal University of Maranhão, São Luís, Maranhão, Brazil; ^4^Federal University of Ceará, Fortaleza, Ceará, Brazil; ^5^Federal University of Maranhão, Imperatriz, Brazil; ^6^Electrical Engineering Department, Federal University of Maranhão, São Luís, Maranhão, Brazil

**Keywords:** epilepsy, seizures, machine learning, ECG, heart rate variability, neural network, data classification

## Abstract

Although electroencephalogram (EEG) is widely used to monitor brain activity in epilepsy, limitations related to the accessibility and reproducibility of measurements may restrict its everyday use. Conversely, wearable methods, easily accessible, such as electrocardiogram (ECG), represent an alternative for indirectly monitoring brain activity through cardiac cycles. A computational model was developed based on statistical cycles and neural networks to measure changes in the morphology of ECG waves. The advantage of this approach over heart rate variability analysis is the detection of brain activity before changes in heart rate occur. In addition, using variance, skewness, and kurtosis centered on the median allowed us to achieve 100% sensitivity, specificity, and accuracy in our analyses, even using less complex algorithms, due to selecting these optimal characteristics. These findings indicate that ECG is a viable, affordable, and effective alternative for estimating epileptic brain activity. This approach’s application of machine learning highlights its potential for non-invasive epilepsy monitoring, providing a cost-effective and accessible solution, especially for vulnerable populations.

## 1 Background

Epilepsy is a neurological disorder that affects approximately 50 million individuals (1) and is characterized by recurrent seizures, manifested as sudden and brief episodes of involuntary movement due to excessive or synchronous neuronal electrical discharges (2). Most epileptic seizures are chronic and effectively controlled with drug therapy (3, 4), although 25% of patients become resistant to the use of anticonvulsants (5). Brain activity in epilepsy is generally classified into four states according to electroencephalogram (EEG) signals: preictal, ictal, interictal, and postictal (6). Epilepsy presents a complex clinical practice, with a significant impact on the quality of life of patients (7). Seizures of sudden onset carry risks such as physical trauma, loss of consciousness, status epilepticus, and sudden death, in addition to contributing to psychological stress and social isolation (8). The cumulative probability of injury in epileptic patients was significantly higher (49% at 12 months and 86% at 24 months) compared to controls (39 and 75%; *p* < 0.0001). However, because 30% of illnesses and 24% of accidents were associated with seizures, excluding these events revealed similar odds of illness and accidents between the two groups (9). The analysis among epileptic patients reveals that nonadherence to treatment was associated with a three-fold higher risk of mortality compared to adherence, as well as a higher incidence of hospitalizations and traffic accidents (10).

Despite technological advances, the diagnosis of epilepsy remains essentially clinical, based on the semiological analysis of the signs, symptoms, and the patient’s history (11). However, patients’ self-reported seizures are unreliable (12, 13), and errors in counting can lead to inappropriate prescription of anticonvulsant medications, either in insufficient or excessive doses (14). The accurate and affordable detection of epileptic seizures still faces significant challenges, mainly due to the reliance on tests such as EEG, which requires specialized training, longer execution time (15) and remains largely inaccessible in many regions of the world, particularly cities with lack of specialized services (16).

The first studies on predicting seizures through EEG records were conducted by Rogowski et al. in 1981 (17). In the late 1990s, Novak et al. (18) used retrospective data, pioneering a method based on time-frequency mapping of R-R interval and autonomous parasympathetic activation to predict seizures several minutes in advance. With the development of research, resource engineering techniques detect seizures with high sensitivity results and low false positive tests but require many manual features that hinder the diagnostic process and practicality (19). Other seizure prediction model options have combined scalp and intracranial EEG, playing a transformative role in the patient’s quality of life, especially in resistant groups, as they allow patients and caregivers to carry out preventive seizure control actions or medication administration (20). Applying machine learning (ML) algorithms to predict and detect seizures has gained significant space due to the advantages cited (21). Techniques such as Support Vector Machines (SVMs) and Artificial Neural Networks (ANNs) have been promising in this domain, with several studies leveraging EEG data to classify and predict seizure events (22–24). EEG has long been the gold standard for monitoring brain activity in epilepsy but is limited by its need for specialized equipment and trained staff in a clinical setting (25). Given the limitations, the potential use of electrocardiogram (ECG) signals as an alternative or complementary approach to seizure prediction has been explored (26). Unlike EEG, ECG is widely accessible, non-invasive, and can be easily integrated into wearable devices, offering a more practical solution for continuous monitoring (27).

Several studies have reported autonomic changes before the onset of a seizure, which are usually reflected in heart rate variability (HRV) parameters, becoming a predictive biomarker of ictal events (28). In temporal lobe epilepsy, studies have detected tachycardia minutes before a preictal event, while some studies have detected bradycardia, illustrating cardiac dysregulation in the face of a seizure (29, 30). By analyzing these subtle changes in HRV, studies have been able to identify early changes of an impending seizure, allowing for precise intervention through single-lead ECG (31, 32).

The time between consecutive ECG heartbeats—the interval between two R-wave (RRI) peaks—is particularly significant in this context, as variations in RRI indicate changes in autonomic function, which are closely linked to brain activity (28). Machine learning models utilizing RRI data have shown promising results in distinguishing between preictal and interictal states, thereby increasing the predictive accuracy of seizure events (25). This capability is crucial for developing practical tools that alert patients and caregivers to an impending seizure, enabling preventative measures to mitigate injury and improve patient safety (5, 33, 34).

Although there are still challenges to its implementation (33, 35), the integration of ECG monitoring into wearable technologies, such as smartwatches and smartphones, represents a significant advance in the management of epilepsy due to greater acceptance by patients (36). Remote measurement technologies (RMTs) equipped with sensors capable of continuously tracking vital signs offer a convenient and scalable solution for real-time health monitoring (35). These devices can collect large amounts of data that, when analyzed using advanced machine learning techniques, can provide personalized insights into a patient’s condition (37). For individuals with epilepsy, this can mean more independence and quality of life (36).

The current study is grounded in the hypothesis of a heart-brain connection, suggesting that subclinical epileptic alterations can influence the cardiac cycle. We aim to explore how accurately ECG signals can estimate such brain epileptic abnormalities, and we hypothesize that ECG could serve as an affordable alternative for assessing epileptic brain activity.

## 2 Methods

**Figure 1:**
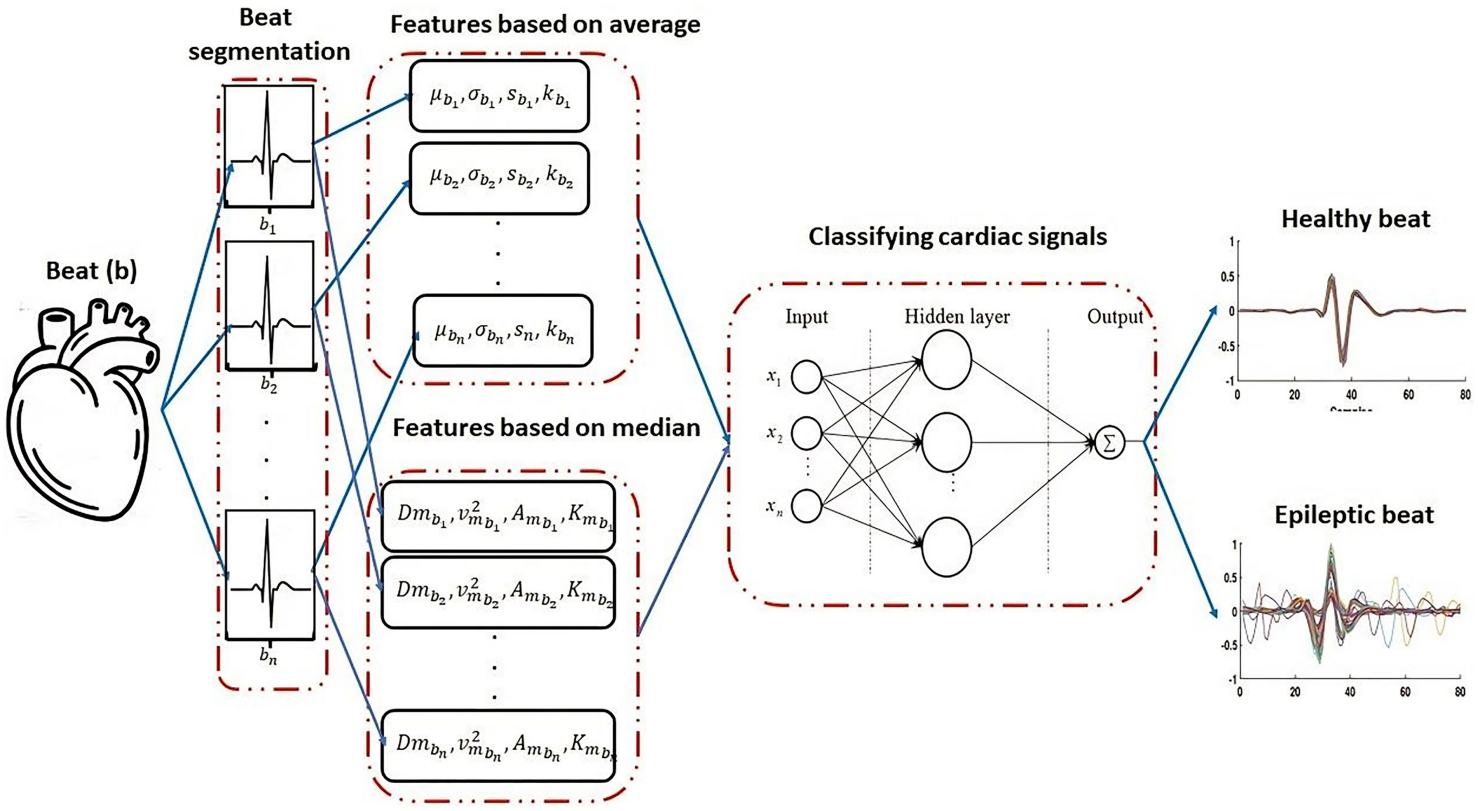
Segmentation of statistical cycles, followed by feature extraction of healthy controls and subjects with epilepsy; two-step feature extraction comprising **(a)** computation of usual statistical features and **(b)** new statistical features based on the median. Computation values are then used as input data in a Multi-Layer Perceptron neural network to classify the statistical cycles between healthy and epilepsy classes.

ECG signals were obtained from the MIT-BIH Normal Sinus Rhythm (NSR) and MIT-BIH Epilepsy Databases. Each ECG signal was segmented into one-second statistical cycles, with characteristics set to 400 ms before and 600 ms after the R wave. The proposed features were compared with the usual features based on the mean for different numbers of neurons in the hidden multi-layer perceptron (MLP) layer. The results were evaluated for sensitivity, specificity, and accuracy based on the algorithm’s performance at the testing stage. The proposed methodology is illustrated in Figure 1.

The MIT–BIH NSR database contains 18 ECG recordings lasting approximately 24 h. The individuals in this database did not have significant arrhythmias: 5 men, aged between 26 and 45 years, and 13 women, aged between 20 and 50 years. Postictal Heart Rate Oscillations in Partial Epilepsy are based on data analysis of 11 partial seizures recorded in five female patients during ECG monitoring. Patients were aged 31 to 48 years, had no clinical evidence of cardiac disease, and had partial seizures with or without secondary generalization of frontal or temporal foci.

### 2.1 Preprocessing

At this stage of the study, we employed the Pan and Tompkins algorithm for QRS complex detection. This algorithm was selected due to its low computational cost and its high accuracy—achieving a 99.3% success rate in detecting QRS complexes in the MIT-BIH Arrhythmia Database (38, 39). The algorithm includes several processing steps: a bandpass filter (comprising low-pass and high-pass filters), a derivative operation, a squaring function, a moving window integrator, and a threshold-based decision rule. The primary objective of this preprocessing step is to minimize the impact of various types of noise and artifacts present in the ECG signal.

To ensure signal clarity, we removed several types of noise, including:

- Power line interference at 60 Hz and its harmonics;
- Baseline wander, a low-frequency noise (0.15–0.3 Hz) caused by respiration, which shifts the ECG baseline;
- Electrode contact noise, resulting from poor contact between the electrode and the skin, which can disrupt signal acquisition;
- Electrode motion artifacts, caused by changes in electrode-skin impedance due to movement;
- Muscle contraction noise, originating from skeletal muscle activity;
- Electrosurgical interference, generated by other medical equipment operating in the 100 Hz to 1 MHz range;
- Instrumentation noise, produced by the electronic components used in ECG acquisition.

To remove these noise components, we followed the methodology described in Pan and Tompkins, applying a bandpass filter to eliminate both low- and high-frequency noise. Specifically, we used a Butterworth filter for this task, which effectively attenuates undesired frequency components while preserving the integrity of the ECG signal.

The ECG signal was amplitude-normalized, and the sampling frequency was set to 256 Hz with a 12-bit resolution over a 10-millivolt range. To avoid measurement artifacts, we excluded the initial and final segments of each ECG recording, corresponding to 1% of the total signal duration. In addition, each ECG signal is defined as:


(1) ECG=(b1,b2,…,bn)

Where bn is the nth cardiac cycle. Each card cycle is defined by bn = {xstart, x2, …, xend}, where x is a heartbeat, xstart, and xend are given by xstart = PR − Fs*λ*, and xend = PR + Fsθ, where PR is the position of the R-peak (PR are found in annotation files in MIT-BIH database), Fs is the sampling frequency and λ and θ are proportion weights of the heartbeat, being λ + θ ≤ 1. xstart and xend are position limits.

### 2.2 Neural network analyses

Many authors perform hyperparameter tuning on classifiers such as Support Vector Machines, Neural Networks, and k-Nearest Neighbors to enhance the accuracy rate. Alternatively, using optimal (or best possible) features is an alternative to achieve higher accuracy rates. Two feature types in the same Neural Network were used to achieve higher accuracy. The first group of features will consist of statistics centered on the mean, including mean, variance, skew, and kurtosis. The second group of features is an adaptation of mean-centric statistics. Unlike classical features that measure the distance from each point to the mean of the data, we propose to use the distance from each point to the median of the data, and we will refer to this distance as the median deviation. Being less sensitive to outliers, the median minimizes noise interference, such as power line signals or muscle activity. This implies that data centralization becomes more homogeneous, resulting in a more concentrated distribution and better data clustering.

#### 2.2.1 Feature extraction

We will use various statistical techniques centered on the mean (mean, variance, skewness, and kurtosis). This variation uses statistics that are no longer centered on the mean of the data but on the median. We will show that this modification is sufficient to increase the hit rate of less complex ANNs.
The new features are based on the median deviation of the data, that is, the distance of each data point to the median (*η*) of the set. The new features are Median Variance ( $\sigma_{\eta }^{2}$ ), Median Standard Deviation ( $\sigma_{\eta }$ ), Median Skewness ( $\psi_{\eta }$ ), and Median Kurtosis ( $\kappa_{\eta }$ ), defined by:
Median Variance ( $\sigma_{\eta }^{2}$ )

(2) $$\sigma_{\eta }^{2}=\frac{\sum_{i=1}^{N}{\left(x_{i}-\eta \right)}^{2}}{N}$$

Median Standard Deviation ( $\sigma_{\eta }$ )

(3) $$\sigma_{\eta }=\sqrt{\sigma_{\eta }^{2}}$$

Median Skewness ( $\psi_{\eta }$ )

(4) $$\;\psi_{\eta }=\frac{\sum_{i=1}^{N}{\left(x_{i}-\eta \right)}^{3}}{\sigma_{\eta }^{3}}$$

Median Kurtosis ( $\kappa_{\eta }$ )

(5) $$\;\kappa_{\eta }=\frac{\sum_{i=1}^{N}{\left(x_{i}-\eta \right)}^{4}}{\sigma_{\eta }^{4}}$$

### 2.3 Classification

We will use a MLP neural network for the classification stage. MLP is one of the most popular and widely used architectures in machine learning applications.

The MLP consists of at least three layers of neurons: an input layer, one or more hidden layers, and an output layer. Each neuron in one layer is connected to all neurons in the next layer. Each connection is associated with a weight that determines the strength of the connection between neurons (40, 41).

MLP is a fully connected neural network, meaning every neuron in one layer is connected to every neuron in the next layer. The input layer receives the input data, the hidden layer processes the information, and the output layer produces the neural network results. Regarding the decision-making process, we aim to demonstrate that choosing optimal features can be an alternative to optimizing MLP.

**Figure 2:**
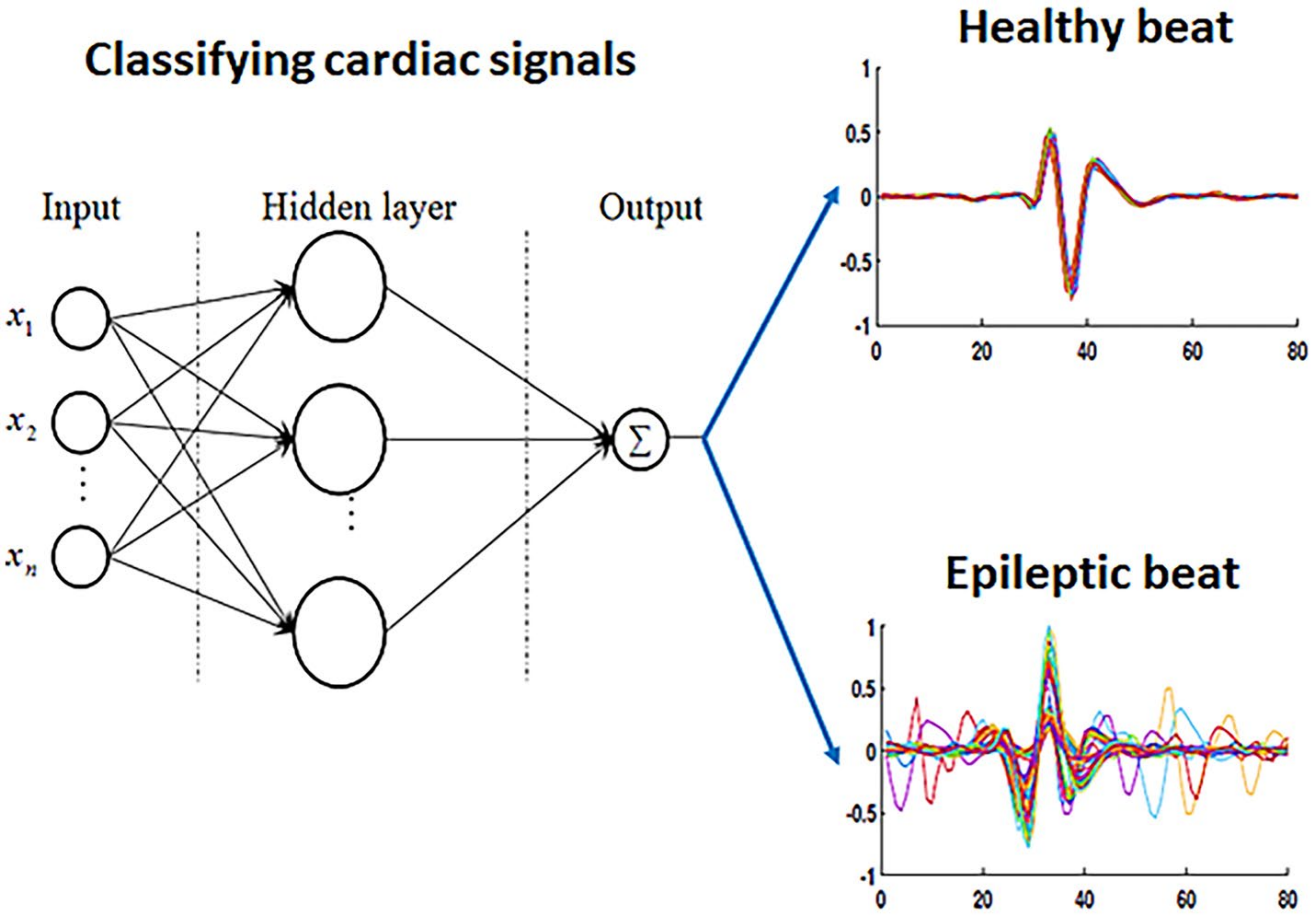
Illustration of the classification stage based on MLP configuration.

Instead of increasing the number of hidden layers, changing activation functions, and optimizing MLP parameters, we will use the most basic MLP configuration. The MLP used in this work consists of an input layer with four inputs, a hidden layer (5, 10, and 100 neurons), and an output layer for two classes (Healthy and Epilepsy). The neural network is trained using the gradient descent training algorithm and employs the sigmoid activation function in all layers (42). Figure 2 illustrates the classification stage.

### 2.4 Evaluation metrics

Evaluation metrics are measures used to evaluate the quality of a machine-learning model or a specific problem-solving solution. They evaluate the model’s ability to generalize and generate accurate results in test data or a production environment.


$$\text{Accuracy}=\frac{\mathrm{VP}+\mathrm{VN}}{\mathrm{PV}+\mathrm{VN}+\mathrm{FN}+\mathrm{FP}},$$

$$\text{Sensitivty}=\frac{\mathrm{VP}}{\mathrm{PV}+\mathrm{FN}},$$

$$\text{Specificity}=\frac{\mathrm{VN}}{\mathrm{VN}+\mathrm{FP}}.$$

Where TP corresponds to the number of true positives for the presence of epileptic seizures, NT the true negatives for the absence of seizures, FP for records with seizures classified as absent, and FN for positives with absence.

To avoid overfitting, the cross-validation technique that evaluates a model’s generalization capacity will be used. Cross-validation partitions the dataset into mutually exclusive subsets and later uses some of these subsets to estimate model parameters (training data), with the remaining subsets (validation or test data) employed in model validation. The K-fold method, with K = 10, was used as a cross-validation technique (43).

## 3 Results

We conducted several experiments to evaluate the proposed methodology. To do this, we used statistical cycles of healthy individuals and statistical cycles of subjects with epilepsy (Equation 1). The experiments were conducted by segmenting the ECG signal into statistical cycles with a constant segment of 1 s, with 400 ms before and 600 ms after the R wave. In total, 2,000 cardiac cycles were used: 1,000 from healthy individuals and 1,000 from individuals with epilepsy. For each cardiac cycle, the mean, variance, skewness, and kurtosis were determined, along with the characteristics proposed in this study, including median variance (Equation 2), median standard deviation (Equation 3), median skewness (Equation 4), and median kurtosis (Equation 5). Training and test data were partitioned by the patient as follows: approximately 70% of the data were allocated for training and 30% for evaluation. We considered 18 recordings from healthy individuals (5 males and 13 females). Among them, three males were assigned to the training set and 2 to the test set. For females, nine records were used for training and 4 for testing.

The hyperparameters used in the MLP implementation include the number of neurons, which were tested separately with values of 5, 10, and 100. The learning rate was set to 0.01, following the default value of the MLP Classifier. The model was trained for 1,000 epochs, using the ReLU activation function (activation = ‘relu’) and the Adam optimization algorithm (solver = ‘adam’). For the loss function, log loss was used, which is appropriate for classification tasks. The stopping criterion was defined as a gradient tolerance of 10^−4^. Regarding the software configurations, the implementation was performed in Python 3.x, using libraries such as NumPy for numerical computations, Pandas for data manipulation, and Scikit-Learn (sklearn) for machine learning models. The primary framework used was Scikit-Learn, specifically the MLP Classifier. The hardware processor carried out for analysis was an 11th generation Intel Core i3 with 4 cores at 3 GHz, with 8 GB of RAM. The execution was performed exclusively on the CPU, without GPU usage. The operating system was Windows 11. Additionally, to increase the robustness of the proposed method, we employed 10-fold cross-validation.

**Figure 3:**
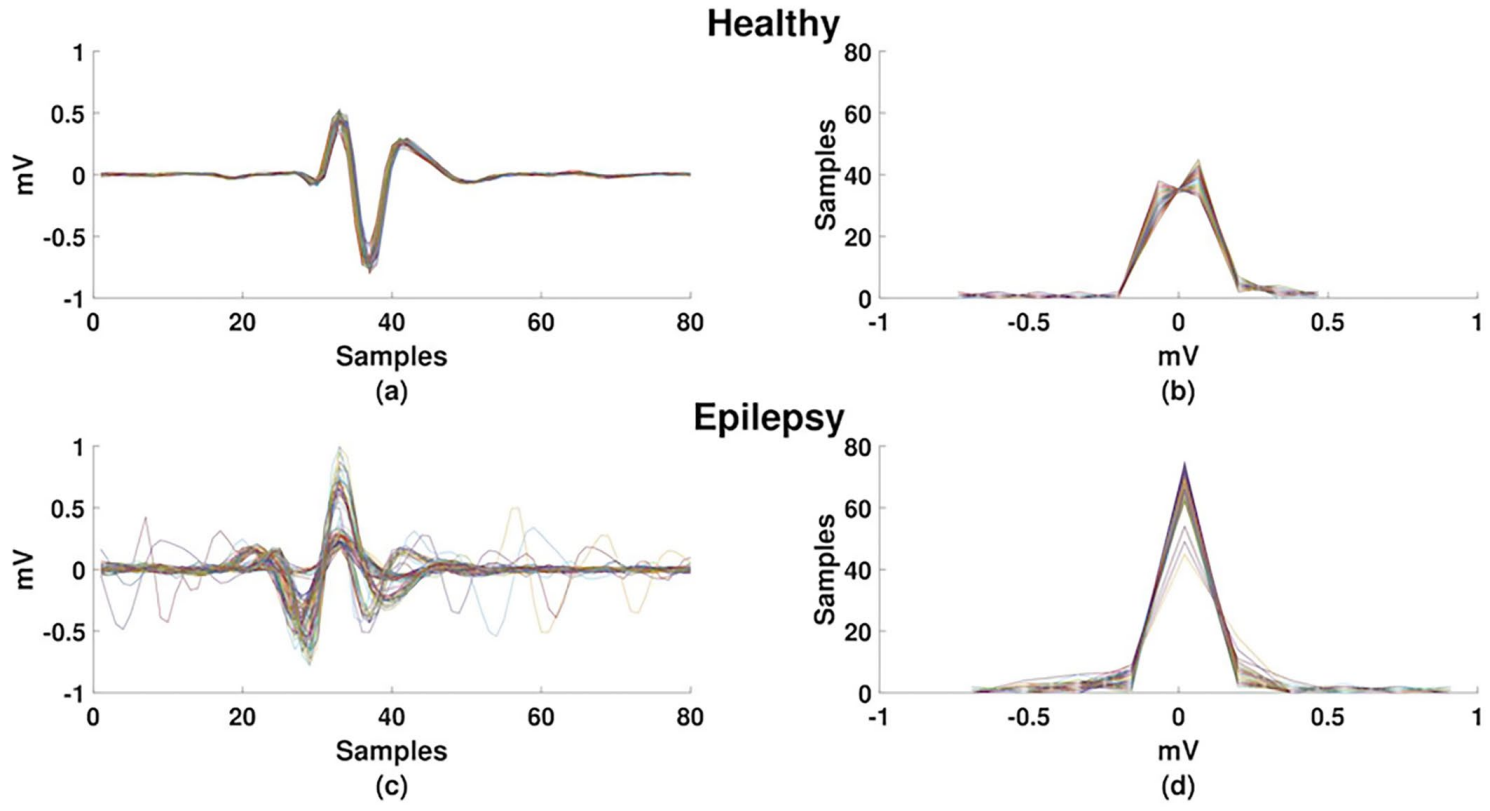
Segmentation of ECG signals in statistical cycles of healthy individuals and subjects with epilepsy. **(a)** Statistical cycles of healthy individuals. **(b)** Distribution of statistical cycles of healthy individuals. **(c)** Statistical cycles of subjects with epilepsy. **(d)** Distribution of statistical cycles of subjects with epilepsy.

In Figure 3, we plot the statistical cycles of healthy individuals and subjects with epilepsy to illustrate the segmentation and deformations of the statistical cycles.

**Figure 4:**
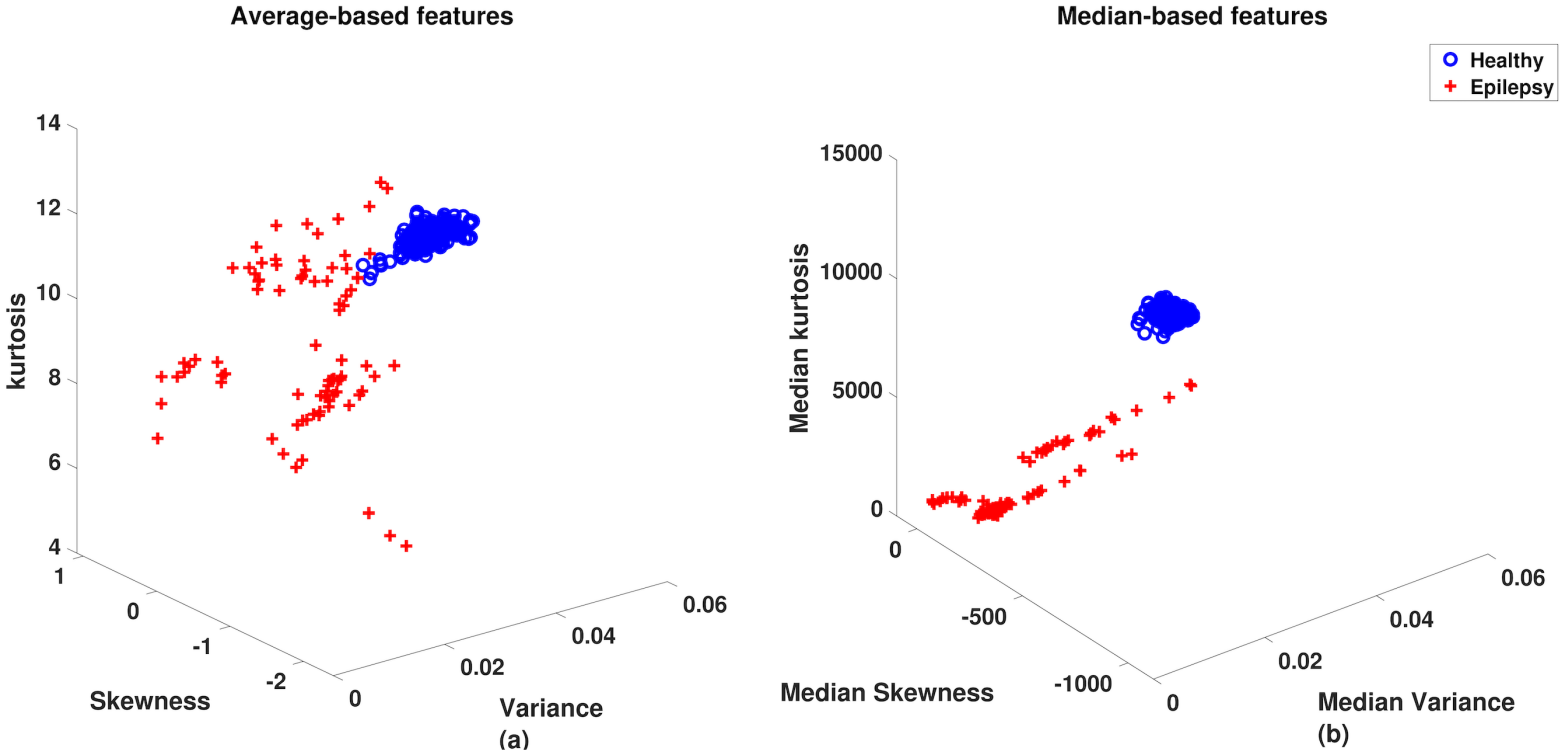
Statistical features of cardiac cycles in healthy individuals and cardiac cycles in individuals with epilepsy: **(a)** Features (Variance, Skewness, and Kurtosis) based on the mean of cardiac cycles in healthy individuals and cardiac cycles in individuals with epilepsy. **(b)** Features (Variance, Skewness, and Kurtosis) based on the median of cardiac cycles in healthy controls and cardiac cycles in individuals with epilepsy.

Two sets of features were used as input to neural networks. The first group consists of classic statistical characteristics based on the mean, such as variance, skewness, and kurtosis, illustrated in Figure 4a. The second group is a proposal of this study, which uses the median of the data as the basis for new statistics called Median Variance (Equation 2), Median Skewness (Equation 4), and Median Kurtosis (Equation 5). These new characteristics are illustrated in Figure 4b.

The characteristics of each healthy cardiac and epilepticus cycle were extracted and plotted on a 3D scatter plot in Figure 4.

The MLP was evaluated with the same input and output neuron numbers (3 and 2, respectively) to test the method’s generality. We vary between 5, 20, and 100 neurons in the middle layer. The results regarding sensitivity, specificity, and accuracy metrics (Table 1) are shown. In addition, the results underwent cross-validation performed in 10 sets, with 70% of the patients for training and 30% for testing.

**Table 1:** Accuracy, sensitivity and specificity values based on the MLP model.

Classifier	Hyperparameters	Accuracy	Sensitivity	Specificity
		Mean (± standard deviation) for *k* = 10 (%)		
Number of neurons		Mean-based method		
MLP	5	0.86 ± 0.03	0.84 ± 0.04	0.87 ± 0.03
	10	0.87 ± 0.02	0.85 ± 0.03	0.88 ± 0.02
	100	0.88 ± 0.02	0.86 ± 0.03	0.89 ± 0.02

Classifier	Hyperparameters	Accuracy	Sensitivity	Specificity
		Mean (± standard deviation) for *k* = 10 (%)		
Number of neurons		Median-based method		
MLP	5	0.95 ± 0.02	0.93 ± 0.02	0.95 ± 0.01
	10	0.96 ± 0.01	0.95 ± 0.01	0.97 ± 0.01
	100	0.98 ± 0.01	0.97 ± 0.01	0.98 ± 0.01

## 4 Discussion

The present study used neural networks to investigate the relationship between brain and cardiac activity by analyzing statistical cycles of ECG signals in individuals with epilepsy and comparing them to healthy controls. The results indicated that the ECG has high sensitivity and specificity to identify statistical patterns in individuals with epilepsy. The epileptic group exhibited more significant variance, skewness, and kurtosis, while the healthy controls showed lower variance and more excellent uniformity in the statistical cycles. Frequency deformations were also observed in the cardiac cycles of individuals with epilepsy, related to the increase in heart rate caused by the autonomic nervous system and deformations in the amplitude of the cycles, which may reflect brain activity.

Specific deformations in the statistical cycles of individuals with epilepsy compared to healthy individuals are demonstrated. There is evidence of a lower number of deformations and homogeneous overlap, with low variation between statistical cycles in the healthy control group (Figures 3a,b) compared to individuals with epilepsy (Figures 3c,d). A frequency deformation can be observed in the statistical cycles of individuals with epilepsy (Figures 3c,d), which is expected due to increased heart rate promoted by the autonomic nervous system. Additionally, it is also possible to observe deformations in the amplitude. These deformations in the cardiac cycles of individuals with epilepsy can be used to estimate brain activity and present an alternative to EEG. Therefore, this study provides evidence of the heart-brain relationship as an option to assess and monitor brain activities in individuals with epilepsy, justifying the heart-brain relationship.

The findings reinforce the heart-brain relationship as a promising tool to assess and monitor brain activity in individuals with epilepsy. When comparing the classic characteristics with those proposed in this study, a significant difference in the dispersion of the data is observed. In Figure 4a, there is a region where the healthy statistical and statistical cycles of individuals with epilepsy overlap, making it difficult to distinguish between classes. However, this overlapping phenomenon is not observed in Figure 4b, allowing for a clear distinction between classes, even for basic MLP configurations. Table 1 states that different MLP configurations can be used to classify healthy statistical and statistical cycles of individuals with epilepsy.

The table demonstrates that optimal features can be an alternative to decrease the number of neurons or hidden layers in a neural network. Two crucial aspects of this work deserve to be highlighted. In conjunction with neural networks, statistical cycles were employed to measure changes in the morphology of ECG waves. One notable advantage of this approach, as compared to heart rate variability (HRV) analysis, lies in its ability to detect brain activity prior to any observable fluctuations in heart rate. This method enables a more proactive and precise assessment of cognitive and physiological responses, offering deeper insights into the mechanisms that drive autonomic regulation. In addition, using variance, skewness, and kurtosis centered on the median enabled us to achieve sensitivity of 97.01%, specificity of 98.01%, and accuracy of 98.01%, in our analyses.

We analyzed the performance of the MLP model by varying the number of neurons in the hidden layer (5, 10, and 100). The “Median-based” method achieved sensitivity of 97.01%, specificity of 98.01%, and accuracy of 98.01% across all neuron configurations, with standard deviation less than 2%, demonstrating robustness against outliers. In contrast, the “Mean-based” method showed sensitivity of 86.03%, specificity of 89.02%, and accuracy of 88.02%, with a standard deviation of approximately 4.04% (Equation 3). As the number of neurons increased, the “Mean-based” method performed better, with more excellent value stability. Additionally, statistical tests (Levene, Student’s *t*-test, and Mann–Whitney) indicated a statistically significant difference (*p* < 0.05) for MLP with 5, 10 and 100 hidden neurons. Despite this, the median-based method is recommended for approximately normal distributions due to its robustness. In contrast, the mean-based method is preferable for normal distributions due to its lower computational cost.

Median-based features were selected for their robustness against outliers, reducing noise interference such as power line signals or muscle activity. Figure 4 illustrates this effect, where data clustering is more evident when using median-based features (Figure 4b) than mean-based features (Figure 4a). Additionally, the median allows the construction of dataset-centered features. This implies that when computing median-based variance, the centralization becomes more homogeneous, resulting in a more concentrated data distribution (Figure 4). If the data are normally distributed (mean ≈ median), the new features will perform similarly to classical ones. However, classical features are recommended due to the additional cost of sorting required to compute the median. On the other hand, for distributions deviating from normality, median-based features are more effective due to their robustness against outliers.

Compared to previous studies that primarily use EEG signals and complex models like CNNs or Random Forests, this study offers a simpler and more accessible approach. It leverages ECG signals—more practical for wearable devices—and introduces median-centered statistical features (variance, skewness, kurtosis), which are more robust to noise and outliers than traditional mean-based metrics. Despite using a basic MLP architecture, the method achieved superior classification performance.

While studies such as Rathod et al. (44) and Mohammadpoory et al. (45) reached high accuracy using EEG and deep learning, they require more computational resources. Others, like Yang et al. (46) combined EEG and ECG but did not explore robust feature engineering. Even simpler models like the MLP used by Nascimento et al. (47) with EEG achieved lower accuracy (94.3%).

Key differences in performance across studies can be explained by: EEG captures brain activity directly but is less feasible for continuous monitoring. ECG, used here, is more practical for real-world, wearable applications; Many studies rely on raw or mean-based features, while this study emphasizes noise-resistant, median-based features.

Ultimately, the proposed method stands out for its balance of simplicity, efficiency, and high accuracy—making it especially suitable for real-time, non-invasive epilepsy monitoring in resource-limited settings.

Table 2 compares the proposed method in this study with approaches found in the literature for epilepsy signal classification.

**Table 2:** Accuracy, sensitivity, and specificity of the proposed method compared to the literature for epilepsy classification.

Author, Year	Classifier	Signal	Accuracy (%)	Sensitivity (%)	Specificity (%)
In this study, 2025	MLP + feature Mean-based	ECG	88.02	86.03	89.02
	MLP + feature Median-based	ECG	98.01	97.01	98.01
Rathod et al., 2025	CNN	EEG	97.55	–	–
Mohammadpoory et al., 2024	Random Forest	EEG	97.98	96.19	99.12
Yang et al., 2022	K-NN	EEG + ECG	96.25	–	–
Aslam et al., 2022	CNN	EEG	–	–	–
Nascimento et al., 2022	MLP	EEG	94.3	90.5	98.1
Cabral et al., 2019	DCC	EEG	96.4	95.5	96.7
Liang et al., 2019	LTCN	EEG	84	99	99
Bhattacharyya and Pachori, 2017	Random Forest	EEG	94.4	97.9	99.5
Fergus et al., 2016	KNNC	EEG	88	88	93
Acharya et al., 2011	SVM	EEG	96	96	97
Shoeb and Guttag, 2010	SVM	EEG	96	–	–

*Current study.

The analysis of Table 2 highlights several important aspects of epilepsy signal classification. The evolution of accuracy over the years is evident, with more recent models (2023–2025) surpassing older studies (<2,020) in metrics such as accuracy and specificity. Model customization and integrating deep learning techniques are evident trends in recent studies, emphasizing progress in the field.

The MLP + Median-based feature method proposed in this study achieved sensitivity of 97.01%, specificity of 98.01%, and accuracy of 98.01%. This result is superior to most listed studies, underscoring the method’s effectiveness. In comparison, other successful methods include Random Forest (45) with 97.98% accuracy and the CNN approach with 97.55% accuracy (48). Although these figures are high, they do not reach the performance of this study. This difference may be attributed to sample variability between our study, Mohammadpoory et al. (45) and Rathod et al. (44). However, it supports our hypothesis that selecting optimal features reduces machine learning complexity, as the MLP used in this study is less complex than the methods used by Mohammadpoory et al. (45) and Rathod et al. (44).

Classical approaches such as SVM (23, 49) and K-NN (46) continue to show solid results but are generally outperformed by more recent methods like CNN (48) and Random Forest (45). Additionally, Yang et al. (46) utilized a combined signal approach, such as EEG + ECG, demonstrating an effort to integrate multiple data sources, which could potentially enhance overall performance.

The literature addresses other possibilities. Jeppesen et al. (37) used a Logistic Regression Machine Learning (LRML) model that was quickly adaptable to each patient, which proved to be superior to a standardized approach. Applying the optimized configuration to patients resulted in a sensitivity of 78.2% and a false alarm rate (FAR) of 0.62/24 h. Compared to the standardized method, there was a 31% reduction in FAR while maintaining similar sensitivity levels. Due to the limited data available from each patient—1–5 seizures recorded each—a low influence on the adaptation of LRML was expected. However, even seizure-free individuals gradually feed the LRML, benefiting from customizing the algorithm’s decision threshold. This way, the portion of the decision threshold based on the non-seizure candidate’s data rapidly changes from an initial generic scenario to a personalized environment, significantly reducing the FAR. Although they make use of a median filter to remove possible false R peak detections, missed R peaks, and/or ectopic heartbeats, the application of skewness, variance, and kurtosis, also centered on the median, can provide a robust and accurate measurement of crisis classification and prediction.

Karasmanoglou et al. (33) developed a method for the early detection of seizures based on a semi-supervised analysis of ECG signals. The study used mean-centric metrics to issue seizure alerts 3–30 min before. The models were evaluated using optimal thresholds, for which the labels “weak” and “hand-selected” were applied to distinguish the analyses. Three anomaly detection techniques were tested: Local Outlier Factor (LOF), Minimum Covariance Determinant (MCD) estimator, and One-Class Support Vector Machines (OCSVM). These techniques were applied to identify anomalous patterns in segments of the RR series of the ECG. The LOF demonstrated a slightly more accurate and consistent alert behavior with accuracy, sensitivity, and specificity of 94.8, 93.0, and 95.8%, respectively, for the “Handpicked,” as well as 93.5, 95.6, and 93.1% for the “Weak.”

Billeci et al. (50) had a median filter to increase the signal-to-noise ratio, obtaining a specificity of 89.34%, a sensitivity of 89.06%, and an accuracy of 88.06% in an ECG database composed of 15 patients with 38 different types of seizures. Ghaempour et al. (32) in addition to the median filter, a high-pass filter allows the passage of signals with parameters higher than a predetermined cut-off point. With this model, they achieved 94.29% accuracy, 100% sensitivity, and 89.47% specificity when analyzing ECG data at 4-s intervals. Behbahani et al. (51) also employed an adaptive decision threshold method to trigger alarms and predict seizures: predictions were made when selected traits exceeded decision thresholds, enabling an average sensitivity of 78.59%. The dataset used for this study was large, consisting of 170 seizures collected from 16 patients during sleep and wakefulness. Our work adds to this growing body of research by providing a comprehensive analysis that integrates cardiac cycle features with neural networks, aiming for high accuracy and practical applicability.

Therefore, the results presented in this study demonstrate the effectiveness of the MLP model in classifying medical data, specifically related to epilepsy diagnosis. Different scenarios were tested, including variations in the number of neurons in the hidden layer and the use of mean- and median-based approaches, providing valuable insights to support medical decision-making in the treatment and monitoring of epilepsy patients. Regarding diagnostic accuracy of 98.01%, the high performance of the model reveals a significant ability to correctly predict the condition of epilepsy patients in most cases. The median-based approach outperformed the mean-based approach, demonstrating greater robustness to outliers, which are common in medical records such as ECG signals. Another essential aspect is the evaluation of sensitivity (recall), which measures the model’s ability to correctly identify positive cases, i.e., patients with epilepsy. High sensitivity implies a low risk of false negatives, ensuring that epilepsy patients are properly identified.

In the tested scenarios, sensitivity increased with the number of neurons, reaching 97.01% in the median-based approach with 100 neurons. Specificity is also a crucial factor, indicating the model’s ability to correctly identify negative cases, i.e., individuals who do not have epilepsy. High specificity reduces false positives, preventing incorrect diagnoses and unnecessary follow-up investigations. The model with 100 neurons using the median-based approach achieved a specificity of 98.01%, an excellent performance for clinical applications. Regarding clinical use and decision support, the model demonstrates usefulness in various epilepsy treatment contexts. High-sensitivity models can be used for initial screening, allowing at-risk patients to receive urgent follow-up. On the other hand, high-specificity models are ideal for confirming diagnoses, reducing the need for invasive or additional tests. The choice between mean- or median-based approaches will depend on the impact of extreme data values, such as fluctuations in ECG recordings. Finally, the results suggest that the MLP model with 100 neurons and a median-based approach is the most effective configuration for minimizing diagnostic errors related to epilepsy. Depending on the clinical context, physicians may choose to prioritize higher sensitivity for screenings or higher specificity for diagnostic confirmations, contributing significantly to a more precise and efficient disease management.

This work can be extended to practical applications in wearable devices through strategies that balance real-time detection with computational efficiency. A promising approach involves the implementation of an optimized embedded system capable of executing the ECG signal processing pipeline directly on the device, reducing the dependence on data transmissions to external servers and minimizing latency. To this end, the median-based feature extraction algorithm can be adapted to use integer operations instead of floating-point operations, reducing energy consumption without significantly compromising accuracy. In addition, model quantization techniques, such as converting the MLP to 8 bits, can reduce memory demand and speed up inferences, making the system viable for low-cost microcontrollers, such as those of the ARM Cortex-M family.

Another practical extension is the development of an adaptive sampling mechanism, where the ECG acquisition rate is dynamically adjusted according to the user’s context. For example, during periods of rest, the system can operate at a reduced sampling rate (e.g., 128 Hz), while in higher-risk situations, such as during sleep or after detecting preliminary anomalies, the rate can be increased to 256 Hz. This approach not only saves power, but also allows the device to prioritize analysis at more clinically relevant times.

Integrating multimodal sensors, such as accelerometers and gyroscopes, can further improve the robustness of the system by ruling out false positives caused by sudden movements or intense physical activity. Contextual data, such as the user’s baseline heart rate and sleep history, can be incorporated into the model to customize detection thresholds, increasing specificity in uncontrolled environments.

To enable large-scale deployment, a hybrid edge-cloud architecture can be adopted. In this scenario, the wearable device performs real-time detection with a simplified model, while raw data is periodically sent to a central server where more complex versions of the algorithm (e.g., LSTMs for seizure prediction) process the information retrospectively. This strategy allows for continuous updates of the model via federated learning, where parameters are refined with data from multiple users without compromising individual privacy.

Finally, validation in real-world settings is crucial to ensure clinical efficacy. Field studies with epileptic patients should assess not only the accuracy of the system, but also its usability, battery life, and user adherence. Partnerships with wearable manufacturers can accelerate technological translation by incorporating the algorithm into commercial smartwatches with ECG sensors already available, such as those from Apple Watch or Fitbit.

In summary, the practical extension of this work requires hardware optimizations (e.g., dedicated processors), algorithmic adaptations (e.g., quantization), and intelligent integration with complementary sensors. These advances would enable continuous, non-invasive and energy-efficient monitoring of epilepsy, democratizing access to early diagnostic tools in clinical and home settings. To ensure greater reproducibility and transparency in current work, the source code publicly is available at the following link.^1^

## 5 Limitations

Our study has limitations that deserve further comments. First the small sample size. Also, in wearable single-lead ECG models, noise and artifacts in the measurement present a difficulty to the sensitivity of the ML (52). An alternative Zambrana-Vinaroz et al. (53) applied was the development of a wearable device that captured ECG, photoplethysmograph (PPG), and ear EEG signals, reaching a sensitivity of 85%, even with the association. However, it has the advantage of being more robust to external disturbances caused by patient movement during detection due to the multiple measurement paths.

Although the features proposed in this study have shown superior results to the classical features for the same neural network configuration, there is a higher computational cost for calculating these new features. This increase in computational cost is due to the median calculation, which is the basis of these new characteristics. For each cardiac cycle, we verify whether the number of elements in the vector containing voltage variations in the cardiac cycle is odd or even before calculating the median, making the proposed method offline, this is one of the main limitations of our work.

One of the primary limitations of our study is the relatively small sample size, which may restrict the statistical power and generalizability of our findings. To address this limitation, future research should aim to include a larger and more diverse sample. This could be achieved through multi-center collaborations, which would allow for broader participant recruitment, or by extending the data collection period to capture a more representative dataset. Such efforts would significantly enhance the robustness, reliability, and external validity of the conclusions drawn.

Other limitations include the absence of a medication regimen and the stratification of epilepsy (moderate, severe). Despite this, kurtosis, skewness, and variance applicability have high potential in medicine, achieving promising results in different fields and basic diagnostic methods, e.g., blood pressure analysis. Populations with scarce features outside major metropolitan areas can benefit from rapid and use-to-use methods.

## 6 Conclusion

This study presents a novel and effective method for detecting epileptic brain activity through ECG signal analysis using neural networks. By introducing median-centered statistical features—variance, skewness, and kurtosis—the proposed approach achieved high classification performance (accuracy: 98.01%, sensitivity: 97.01%, specificity: 98.01%) even with a simple MLP architecture. These results highlight the strength of robust feature engineering over model complexity.

A key contribution of this work is demonstrating that ECG, a widely accessible and non-invasive signal, can serve as a reliable proxy for brain activity monitoring in epilepsy. Unlike EEG, which requires specialized equipment and clinical settings, ECG can be easily integrated into wearable devices, enabling continuous, real-time monitoring.

The clinical potential of this method is significant. Its implementation in smartwatches or portable ECG monitors could allow for early seizure detection, providing timely alerts to patients and caregivers. This capability not only enhances patient safety by reducing the risk of injury but also supports more personalized and proactive epilepsy management. Furthermore, the method’s low computational cost makes it suitable for deployment in low-resource settings, contributing to more equitable access to neurological care.

In summary, this study advances the field of epilepsy monitoring by offering a cost-effective, scalable, and clinically relevant solution. Future research will focus on validating the model in real-world wearable systems and expanding its application to other neurological conditions.

## Data Availability

The raw data supporting the conclusions of this article will be made available by the authors, without undue reservation.

## Footnotes

^1^ https://drive.google.com/file/d/1B5eMZVeI6Bi8jtopNEg3zq35JfxPIsNp/view?usp=drive_link

## Glossary

EEG: electroencephalogram
ECG: electrocardiogram
ML: machine learning
SVMs: support vector machines
ANNs: artificial neural networks
HRV: heart rate variability
RRI: interval between two R-wave
RMTs: remote measurement technologies
NSR: normal sinus rhythm
MLP: multi-layer perceptron
TP: true positive
NT: true negative
FP: records with seizures classified as absent
FN: positives with absence
LRML: logistic regression machine learning
FAR: false alarm rate
LOF: local outlier factor
MCD: minimum covariance determinant
OCSVM: one-class support vector machines
PPG: photoplethysmograph
