# Supplementary material for: Neural network-based method for measuring the impacts of epileptic brain activities on cardiac cycles
Source: Front Neurol. 2025 Jul 30;16:1555162. doi: 10.3389/fneur.2025.1555162 (PMC12351131; doi:10.3389/fneur.2025.1555162)
Supplement: Supplementary file 1 [file Table_1.DOCX]

Table. Accuracy values based on MLP model

|  | Neurons in the hidden layer | Sensitivity | Specificity  (%) | Accuracy |
| --- | --- | --- | --- | --- |
|  | 5 | 84.2 | 86.7 | 85.6 |
| Average-based | 10 | 94.55 | 94.55 | 94.55 |
|  | 100 | 100 | 100 | 100 |
|  | 5 | 100 | 100 | 100 |
| Median-based | 10 | 100 | 100 | 100 |
|  | 100 | 100 | 100 | 100 |
